# From River Blindness to Neglected Tropical Diseases—Lessons Learned in Africa for Programme Implementation and Expansion by the Non-governmental Partners

**DOI:** 10.1371/journal.pntd.0003506

**Published:** 2015-05-14

**Authors:** Catherine Cross, Franca Olamiju, Frank Richards, Simon Bush, Adrian Hopkins, Danny Haddad

**Affiliations:** 1 Sightsavers, London, England, United Kingdom; 2 Mission to Save the Helpless (MITOSATH), Jos, Nigeria; 3 The Carter Center, Atlanta, Georgia, United States of America; 4 Sightsavers, Accra, Ghana; 5 Mectizan Donation Programme, Atlanta, Georgia, United States of America; 6 Emory Global Vision Initiative, Atlanta, Georgia, United States of America; University of South Florida, UNITED STATES

## Introduction

After more than 20 years of action against some of the most debilitating neglected tropical diseases (NTDs), lessons have been learned by the non-governmental development organisations (NGDOs) in the light of changes in programme strategies and partnerships. This article aims to summarise the development of the non-governmental networks supporting the NTD programmes, starting with the original 1992 model to combat onchocerciasis (river blindness), and will review the lessons learned that have equipped the NGDOs to step up their support to NTD control and elimination.

At the beginning of the 1990s, a small group of seven NGDOs began to work together to support onchocerciasis control [1]. Today, more than 50 international and national NGDOs (of which 30 collaborate at the international level) work together to control or eliminate five priority NTDs affecting more than one billion of the poorest people [2]. This is a significant contribution to the objectives of World Health Assembly resolutions and the London Declaration on NTDs [3], as well as addressing Millennium Development Goal (MDG) 1, “Eradicate extreme poverty and hunger;” MDG 6 “Combat HIV/AIDS, malaria, and other diseases;” and MDG 8, “Develop a global partnership for development.” In 2013, the first annual report of the London Declaration on Neglected Tropical Diseases recorded increased treatments and funding and significant progress towards the World Health Organization (WHO)’s roadmap for implementation for control, elimination, or eradication by 2020.

This achievement has been made possible by developing networks of NGDOs and partnerships with governments of the endemic countries, with international and bilateral agencies, with drug donation programmes for specific diseases, and with the communities affected by those diseases [4]. By 2011, NGDOs reported support in 125 countries to more than 330 million treatments for five priority diseases: onchocerciasis, trachoma, lymphatic filariasis (LF), schistosomiasis, and soil-transmitted helminthiasis (STH) [5]. These diseases have effective mass drug administration (MDA) strategies supported by donated or very-low-cost drugs available to control or eliminate them [6,7].

## Development of the NGDO Networks—The Original Model

The original model was the NGDO Coordination Group for Onchocerciasis Control, formed in 1992 in response to a debilitating and blinding disease affecting large parts of Africa where at least 102 million people are estimated to be at high risk [8]. Control was accelerated and expanded after the arrival of the drug Mectizan (ivermectin) in 1987, when it was registered for human use by Merck & Co., Inc. Later the same year, Merck made the ground-breaking announcement of the donation of Mectizan with the goal of making it available, free of charge, as much as needed and for as long as needed, for the elimination of onchocerciasis as a public health problem in all endemic countries [9]. The story of onchocerciasis control with ivermectin [10–13] and the more recent moves towards elimination in parts of Africa [14,15] have been well documented. The role played by NGDOs in developing the community methodology needed to ensure long-term treatment has also been described [16], as has their role within the African Programme for Onchocerciasis Control (APOC) [17,18]. It is not proposed to re-trace these developments in detail here, but to identify the lessons learned in moving from a successful single disease control programme to a partnership tackling multiple diseases requiring not just mass drug administration (MDA) but in some cases surgery (e.g., trachoma, lymphatic filariasis) and behavioural and environmental change (e.g., trachoma, schistosomiasis, and STH).

A key relationship for the NGDOs is with the WHO. The International Agency for the Prevention of Blindness (IAPB) had forged and maintained a strong relationship with the WHO Programme for Prevention of Blindness and Deafness since the 1970s [19]. Five IAPB NGDO members—Christoffel-Blindenmission (CBM), Helen Keller International (HKI), International Eye Foundation, Organisation pour la Prévention de la Cécité, and Sightsavers—became active participants in onchocerciasis control in the early 1990s forming the NGDO Coordination Group for Onchocerciasis Control in 1992 with Africare and, importantly, with the River Blindness Foundation (RBF), which brought significant funding for implementation activities. The partnership developed with WHO at various levels—at headquarters in Geneva, with the Africa Regional Office, and with the West Africa Onchocerciasis Control Programme (OCP). The existence of a formal grouping, with a secretariat at WHO Geneva that was part-funded by the NGDOs, conferred legitimacy on their work and gave them a voice on programme issues. From these partnerships sprang APOC, the aim of which was to expand ivermectin distribution to cover all remaining endemic areas in Africa and which benefited from the established OCP structure and the World Bank funding mechanism. APOC also benefited from the funds NGDOs brought in to the programme and from their field activities prior to APOC’s establishment in 1995, as scaling up was achieved quickly, especially in Nigeria, a country with the largest number of onchocerciasis sufferers. While NGDOs were originally seen as supporting programmes at field level, their role in the establishment of APOC eventually assured their representation on all of APOC’s governing and advisory bodies. Twenty years later, NGDOs sit at the table alongside WHO, the World Bank, the national programmes, the donors to those programmes, the pharmaceutical companies, and their donation mechanisms.

The work of the NGDOs involved in these networks could not have been achieved without a close relationship with other key players, including donors of resources and medicines. Drug donations made by major pharmaceutical companies are channelled through mechanisms that have been set up to manage them. The first such was the Mectizan Donation Program (Merck), which was established to manage the donation of ivermectin. In the early 1990s, Mectizan Donation Program (MDP) formed a working relationship with the NGDOs supporting onchocerciasis control. Subsequently the Albendazole Donation Programme (GlaxoSmithKline), the International Trachoma Initiative (Pfizer), and Children Without Worms (Johnson & Johnson and GlaxoSmithKline) were established to manage the donation of other NTD drugs. These donation programmes provide the drugs free of charge to endemic countries, a very significant contribution to the cost of implementing NTD activities, as well as technical and often additional financial resources to address specific problems encountered on the ground.

## Development of Subsequent NTD Networks

**Fig 1:**
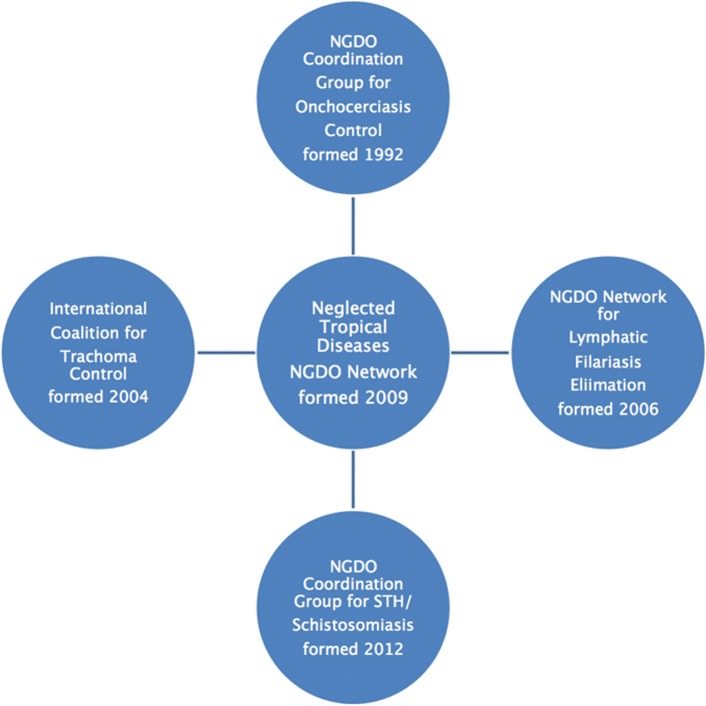
Neglected tropical diseases NGDO network 2013.

Working within APOC from 1995, the NGDO Coordination Group consolidated its partnership and proved its usefulness as a model for other groups working on NTD control programmes. In the following decade, four other partnerships developed. Three are devoted to specific diseases and the fourth is a loosely organised umbrella network designed to provide an overview and maintain coordination (see Fig 1).

Over that time, NTDs began to receive increased attention, recognizing that a number of the diseases are susceptible to control and even elimination using drugs that are either donated or cheap on the open market. Sustained advocacy by prominent members of the scientific community successfully drew international attention to the health benefits and relatively low cost of NTD control and, where possible, elimination [6,7].

In 1998, when GlaxoSmithKline (GSK) decided to donate albendazole, which, combined with ivermectin, became the tool for elimination of lymphatic filariasis in Africa, GSK did so in coordination with MDP. Rather than set up a separate scientific oversight committee, GSK worked with the MDP to share the Mectizan Expert Committee, which was renamed the Mectizan Expert Committee and Albendazole Coordination (MECAC). This mechanism manages both drug donation programmes. As MDP had long been a member of the NGDO Coordination Group for Onchocerciasis Control, so the opportunities for NGDOs to become involved in LF became obvious. The NGDO Network for LF Elimination was established in 2004 after the formation in 2000 of the Global Alliance to Eliminate LF, a partnership that supports WHO’s objectives of eliminating LF as a public health problem by 2020 and preventing and alleviating disability and suffering of those already infected [20]. Unlike onchocerciasis control, which is about the delivery of ivermectin tablets maintaining high coverage for 20 years or more, LF elimination can be achieved in a shorter timescale but leaves its sufferers behind with chronic conditions such as lymphoedema. While the thrust of the NGDO Coordination Group for Onchocerciasis Control was to support programmes in Africa (as well as to completely eliminate the disease in the Americas and Yemen), 65% of those affected by LF live in Asia. The NGDO Network for LF Elimination therefore needed to engage with organisations working in Asia and those with an interest in morbidity management, such as Health and Development International (HDI) and Handicap International (which, indeed, chaired the initial meetings of the Network). The LF network not only has the challenge of scaling up MDA but also of promoting morbidity control and hydrocele surgery.

Trachoma was recognized by the League of Nations as the principal cause of blindness in tropical countries as early as the 1930s [21]. Subsequently, efforts were made to control it using tetracycline eye ointment and eyelid surgery, and NGDO-supported eye care programmes included topical treatment of active trachoma and lid surgery. However, in the 1990s there was a renewed effort to eliminate blinding trachoma with the adoption in 1996 of the WHO-recommended SAFE strategy (Surgery, Antibiotics, Facial cleanliness, Environmental improvement) [22]. In 1997 the first meeting of the WHO Alliance for the Global Elimination of Blinding Trachoma by the year 2020 (GET 2020) took place. The Alliance was formed of national health programmes in trachoma-affected countries, donors to those programmes, representatives of the pharmaceutical company Pfizer Inc., the scientific community, WHO, and NGDOs. A year later, the International Trachoma Initiative (ITI) was set up as a channel for the new donation of Zithromax (azithromycin) by Pfizer Inc., a safe, effective and more useable drug than tetracycline. The annual meeting of the Alliance continues to take place, hosted by the WHO. It is an essential forum for discussing all aspects of trachoma elimination but too large and formal a meeting for the NGDOs to address issues such as coordination, resource mobilization, and the need to engage with water and sanitation agencies. After a number of brief informal meetings in 2004, a trachoma-specific NGDO network was established, the International Coalition for Trachoma Control (ICTC), with the objectives of data collection, sharing information, and coordination, advocacy, and resource mobilization in order to promote the SAFE strategy. In 2011, the ICTC developed a global roadmap, the 2020 Insight plan, to advocate for implementation of the full SAFE strategy towards the goal of elimination by the end of the decade. This has eventually led to the achievement of significant funding for a global trachoma mapping project and SAFE implementation.

Other parasitic diseases also began to be addressed, as when in 2002 the Bill & Melinda Gates Foundation's Global Health Program granted an award to establish the Schistosomiasis Control Initiative based at Imperial College, London. The award was directed to delivering 40 million treatments of praziquantel for schistosomiasis control in Africa. Then in 2006, Johnson & Johnson set up the Mebendazole Donation Initiative, now known as Children Without Worms (CWW), initially donating 50 million treatments annually for children in endemic countries suffering from soil-transmitted helminths. This increased to 200 million treatments per year by 2010. In October 2010, GSK made another major commitment to NTD control when they announced the donation of 400 million treatments of albendazole for school children yearly, in addition to the 600 million tablets they were already donating annually for LF elimination. A variety of other organizations use generic medicines purchased at low cost to support de-worming programmes, including generous donations by UNICEF. Support to schistosomiasis and STH control programmes from a number of sources raises issues of coordination, and NGDO partners have now formed the NGDO Coordination Group for Soil-transmitted Helminthiasis and Schistosomiasis. Members include not only those working directly on disease control but also those focusing on school health, nutrition, hygiene education, and water and sanitation. The goal is to promote a comprehensive approach to tackling these diseases and to break the cycle of reinfection.

There were now four international groupings of non-governmental agencies working on priority NTDs. In some cases, the same people represent their organisation at the international level and can achieve an overview of activities in all NTDs and identify where the work of their agency can complement that of other NGDOs. However, this is not always the case—and not always desirable since different expertise is needed for different diseases—and coordination becomes a challenge. A measure of coordination is achieved by the fact that three of the major drug donation programmes (MDP, ITI, CWW) are hosted within the Task Force for Global Health, Atlanta, United States, and they work closely together and with the supporting NGDOs. However, recognising the need for greater coordination within the increasingly complex world of NTD control, the NGDO groups addressing onchocerciasis, LF, and trachoma began to meet together, and in September 2009, it was agreed to form the NTD NGDO Network [2] to focus on issues of co-implementation and scale-up, while the disease-specific groups concentrate on the technical aspects of control and elimination. Most recently, the 14 NGDOs that in 1966 formed the International Federation of Anti-Leprosy Associations have seen the value of joining the NTD NGDO Network. Table 1 lists the current membership of the NTD NGDO Network.

**Table 1:** Members of the neglected tropical diseases NGDO network 2013.

Charitable Society for Social Welfare, Yemen	Organisation pour la Prévention de la Cécité
Centre for Neglected Tropical Diseases, Liverpool	RTI International Envision
CBM	Schistosomiasis Control Initiative
Fred Hollows Foundation	Sightsavers
Handicap International	The Carter Center
Health and Development International	Ulls del Mon
Helen Keller International	United Front Against River Blindness
IMA World Health	United States Fund for UNICEF
International Federation of Anti-Leprosy Associations	University of Melbourne Indigenous Eye Health Program
International Trachoma Initiative	Wilmer Eye Institute at Johns Hopkins
Kilimanjaro Centre for Community Ophthalmology	World Vision International
Lepra	**Associate members**
Light for the World	Children without Worms
Lions Club International Foundation	Glaxo Smith Kline
London School of Hygiene and Tropical Medicine	International Agency for the Prevention of Blindness
Malaria Consortium	Johnson & Johnson
Mectizan Donation Program	Merck and Co Inc.
MITOSATH	Pfizer Inc.
Orbis International	

## Lessons Learned

The NGDOs working in NTDs have the common desire to support national programmes to control and, if possible, eliminate these endemic diseases, recognizing that those suffering are among the “bottom billion” [23], the poorest people in the world. Their commitment is articulated in their support to the London Declaration on Neglected Tropical Diseases through the Sydney Communiqué of September 2012 [2]. NGDO input consists of financial and technical support to training, programme strategy and implementation, operational research, and production of health education materials and management manuals.

The main lessons learned are the need for good coordination, joint approaches to programme management, and the development of partnerships. Flexibility in the use of funds and in adapting traditional approaches, as well as advocacy and support to research and development, have also been key lessons for the NGDOs. These are discussed in the following sections.

## Coordination

Coordination has become increasingly important in moving from the delivery of tablets aimed at a single disease to multiple diseases whose control depends upon important, non-tablet components. The original model, with an officer part-funded by NGDOs in WHO Geneva, was critical in establishing the bona fides of organisations still untested in MDA, and the post was also able to act as neutral broker when necessary. As APOC developed the systems set up to manage a major international health programme with high visibility and acceptance by the recipient countries, while far from the more flexible mode of working of NGDOs, it provided a framework and a discipline not just for the national programmes but also for the non-governmental partners. At the same time, APOC benefited from the greater flexibility of NGDOs—from time to time they pre-finance a programme while APOC’s procedures are being finalised. APOC was also able to report significant treatment figures early on, especially in Nigeria with its high burden of disease, because of the pre-APOC support to national programmes by the NGDOs.

The need for coordination of NGDO effort was first learned in the beginning of the 1990s in Nigeria. At the time, agencies were providing fragmented support to state and local government area programmes—sometimes with more than one NGDO in the same state. The decision to move towards “one state, one NGDO” greatly improved efficiency and accountability, as did the setting up of a Nigerian NGDO coalition to work with the National Onchocerciasis Task Force. This model has been replicated throughout the APOC programmes, and the lesson learned is that geographic coordination of NGDO support is necessary for maximum efficiency. Effective coordination is a key consideration within subsequent NTD programmes in order to ensure high geographic coverage of all endemic areas.

## Programme Management and the Development of Partnerships

Trachoma and lymphatic filariasis require a multi-sectorial approach, and technical coordination remains a challenge. Few agencies had prior expertise to take on the full SAFE strategy (trachoma) or support MDA and morbidity management (LF), and MDA moves at a relatively fast pace, while clearing the backlog of surgery or improving sanitation are slower, more costly, and continue to prove a challenge. The onchocerciasis programmes taught NGDOs the need to work closely together in programme management and in the support to training of district and community personnel. When in the early 1990s a consortium of European agencies received funding from the European Union (EU) to begin ivermectin distribution in five countries, the agencies largely operated independently and did not collect standardized data, and this proved to be a problem when the “lead agency” was required to report to the EU. However, over the past 20 years, NGDOs have got to know each other better and have learned to achieve a degree of integration of their systems, when appropriate.

Local NGDOs and community groups have become involved in NTD programmes in several countries. In Nigeria and Liberia, for example, Mission to Save the Helpless (MITOSATH) and the Christian Health Association of Liberia have respectively formed partnerships with international NGDOs and not only support programme implementation but also perform an important function of advocacy within the country. MITOSATH also works at the international level within the NTD NGDO Network.

The need to develop new partnerships becomes critical once the implementation strategy requires more than MDA. In trachoma control, for instance, prevention of blindness agencies could handle drug distribution, lid surgery and health education, but had no expertise in water and sanitation. A major trachoma control programme in Kenya was successful in obtaining EU funds through working closely with the United Kingdom–funded school building programme and with Amref Health Africa, a well-established NGDO with expertise in water and sanitation, but one outside of the group familiar to prevention of blindness agencies. More recently the ICTC partnership has obtained a grant of GBP 42.8 million from the Queen Elizabeth Diamond Jubilee Trust to scale up trachoma interventions in Commonwealth countries. The programme, with funds channelled through Sightsavers, aims to reduce the trichiasis surgical backlog, support MDA, raise awareness of hygiene, particularly the need for face washing of children, and initiate measures to improve the environment, including better sanitation and water supplies.

The lessons learned are the need for strategic partnerships to address all components of NTD programmes and the importance of adopting clear strategies for programme development. Nevertheless, this is not easy—NGDOs have different mandates, work cultures, and support constituencies and often are in competition with each other for financial resources. Greater availability of funding has brought new partners to the NTD table and educating them and establishing strong partnerships require continuous efforts.

## Flexibility and Funding

Flexibility in NGDOs’ traditional, sectorial approaches to programming is also needed. In Nigeria, The Carter Center (TCC) pioneered the grafting of other NTD activities onto established onchocerciasis MDA programmes, including LF elimination and schistosomiasis control [24]. Prevention of blindness NGDOs used the community networks established by onchocerciasis control to introduce other programmes, such as cataract case-finding and Vitamin A supplementation, in collaboration with government, and this made a significant contribution to the strengthening of the health system [25].

Some NGDOs have departed from their original mandate in order to support integrated NTD programmes. The Schistosomiasis Control Initiative has extended its remit to address other NTDs. In the case of Sightsavers, a major decision had to be taken in 2009 by Sightsavers’ Council to move away from 60 years of exclusive focus on blindness, and the organisation is now able to support all components of NTD programmes.

Flexibility is also required in funding. Right at the beginning of onchocerciasis control using ivermectin, it was known that these were not short-term programmes but would have to be sustained for at least 10–12 years. With greater understanding of the effect of ivermectin, this became 20 years or more, depending on endemicity and coverage. Fragmented funding has therefore been a problem for NGDOs (as for all APOC partners). NGDOs raise funds from a variety of sources and some are not able to plan more than a year ahead. A lesson learned in the past 20 years is that in some instances, co-funding or even takeovers are necessary in order to safeguard existing programmes. In Tanzania, Sightsavers was able to take over funding of areas that IMA Worldhealth could no longer support. In Nigeria, CBM mentored local NGDO MITOSATH in Taraba State and, in South Sudan, took over the programme previously supported by HealthNet International. More recently, Sightsavers has provided assistance to a smaller NGDO, United Front Against River Blindness (UFAR), dedicated to onchocerciasis control in the Democratic Republic of Congo.

The availability of funding has eased with new interest in NTD control and elimination by major donors such as USAID and the UK Department for International Development (DFID) working together to complement each other’s contributions. For example, DFID has granted GBP 9.5 million to UNITED, a consortium of agencies including five NGDOs, with the aim of strengthening integrated NTD control activities in three northern states of Nigeria, while USAID focuses support to Nigeria’s NTD programmes in the southern states and at central ministerial level. Nevertheless, NGDOs still have to find a proportion of the support needed from their own sources. Uncertain funding has taught NGDOs the need for flexibility in order to sustain NTD programmes.

## Advocacy

At national and international level NGDOs see their role as advocates for the disease control programmes—to encourage other agencies to participate, ensure that high coverage is achieved and maintained throughout the endemic areas and that all programme components are addressed.

Advocacy has played a large part in the actions of the various NGDO groups. The first and best known is by Sightsavers’ founders, Sir John and Lady Wilson, who are credited with identifying that onchocerciasis was responsible for the high numbers of blind people in West Africa and with calling the disease “river blindness” [26]. When ivermectin became available, the concept of preventing children in Africa from going blind through an annual treatment with the drug was so appealing that in the early 1990s a children’s TV programme in the UK raised over GBP 1M. The RBF was set up in the US specifically to promote support to the control of onchocerciasis, and their work was subsequently taken over and expanded by TCC, as former president Carter has long been an important advocate for NTDs.

Advocacy efforts initially fell on stony ground when the trachoma NGDOs tried to engage some of the major water and sanitation agencies (although World Vision in West Africa was an exception). The much higher cost of these activities, the already huge demand for clean water throughout low-income countries, and the lack of additional funding available were barriers to engaging specialist agencies to support the development of the “E” component of SAFE. However, in the last decade, high-profile advocacy by members of the scientific community has led to increased availability of funding such as the USAID donation of US$400 million, through RTI and FHI 360, and UK government grant of GBP250 million, through Schistosomiasis Control Initiative (SCI) and Liverpool Centre for Neglected Tropical Diseases (CNTD), along with an additional GBP10 million, through Sightsavers, to the Global Trachoma Mapping project, which aims to map all remaining suspected endemic districts by March 2015. This higher profile has also attracted new players to the scene, both those involved in programme implementation and grant-givers such as the Queen Elizabeth Diamond Jubilee Trust.

The role of advocacy and engaging the support of prominent people have been essential lessons in making the case for the control of obscure diseases largely unknown in the West.

## Research and Development

Much of the earliest research into onchocerciasis and its effects on the human population in West Africa was sponsored by NGDOs [27]. Recently, lessons learned from NGDO- supported research projects have contributed to programme strategy, such as studies undertaken by TCC on the use of traditional kinship systems in onchocerciasis control in Uganda [28] and the collateral impact of onchocerciasis MDA on LF and STH [29].

NGDO staff members have also brought important learning into their organisations through their participation in major research projects, such as the multi-country study on community- directed interventions [30]. The process of learning has fed into programme implementation through the development of health education materials and manuals by several agencies.

The manual *Implementing the SAFE strategy for trachoma control* published jointly by TCC and ITI [31] has become the key resource for managing the F and E components of the SAFE strategy, while ICTC has reviewed practices in surgery and azithromycin distribution that have been published as manuals sponsored by the partnership.

## Conclusion

Programmes addressing individual NTDs are increasingly working together and collaborating with other health and development activities, thus contributing towards health sector strengthening as well as disease control and elimination. Experience has shown the importance of establishing multi-sectorial partnerships because individual NGDOs rarely wield much decision-making power, especially at an international level. By coordinating their work in advocacy, programme management, and access to funding, NGDOs have been able to learn from each other, expand their activities, and adopt flexible approaches to programme support. In 2013, the NGDOs working on onchocerciasis acknowledged the shift needed from control to elimination by changing the name of the group to the NGDO Coordination Group for Onchocerciasis Elimination. To reach NTD elimination targets in Africa, further coordination and the development of integrated approaches will be required. NGDOs will need to collaborate closely in order to expand support to neglected tropical diseases programmes, drawing on the lessons learned over more than 20 years.
